# Reducing missed opportunities for vaccination in selected provinces of Mozambique: A study protocol

**DOI:** 10.12688/gatesopenres.12761.1

**Published:** 2017-11-06

**Authors:** Bvudzai Priscilla Magadzire, Gabriel Joao, Stephanie Shendale, Ikechukwu Udo Ogbuanu

**Affiliations:** 1 VillageReach, Seattle, WA, USA; 2 VillageReach, Maputo, Mozambique; 3 Expanded Programme on Immunization (EPI) Department of Immunization, Vaccines and Biologicals (IVB), World Health Organization, Geneva, Switzerland

**Keywords:** missed opportunities for vaccination, immunization, Mozambique, LMIC

## Abstract

**Background**: A missed opportunity for vaccination (MOV) refers to any contact with health services by an individual who is eligible for vaccination, which does not result in the person receiving the vaccine doses for which he or she is eligible. A consortium of partners, including VillageReach, the Ministry of Health in Mozambique and the World Health Organization, will implement a strategy to reduce MOV in Mozambique. The strategy involves demonstrating the magnitude of missed opportunities and their causes, and exploring tailored health system interventions to reduce them, with the aim of increasing vaccination coverage and timeliness of vaccinations.

**Methods**: A mixed-methods approach will incorporate both quantitative and qualitative tools. The assessment will target caregivers of children between the ages of 0–23 months who attend a health facility in the selected districts on the day of the assessment. Caregivers who are at least 18 years old will be eligible for inclusion. Another component of the assessment will target all health workers in the selected health facilities on the day of the assessment. A sample of 30 health facilities in different regions of the country will be assessed, with a target sample size of 600 caregiver exit interviews, 300 health worker interviews and focus group discussions with both caregivers and health workers. Data collection will commence late 2017, and the data will be electronically captured, managed and analyzed. Thematic analysis of data from the qualitative aspects of the assessment will be conducted, presenting the scope of interviews, representative verbatim quotes and key conclusions.

**Conclusions**: A concerted effort to reduce or eliminate MOV could increase vaccine coverage by up to 30% and may contribute to wider improvements in efficiencies of service delivery beyond the immunization program. In addition, the findings could contribute to a better understanding of MOV in similar settings.

## Introduction

According to a report by the World Health Organization (WHO), global vaccination coverage has stagnated below the 90% target for more than a decade, meaning that a significant proportion of the global annual birth cohort remains unvaccinated with DTP3. Similar to many other developing countries, Mozambique has experienced challenges with increasing immunization coverage. The recently identified cVDPV polio case and pockets of unvaccinated children in one province indicates an urgency to strengthen the Expanded Program on Immunization (EPI). There is a growing need to understand the persisting barriers and the degree to which these issues affect eligible children who could potentially be vaccinated in the absence of other challenges. Thus, a consortium of partners, including VillageReach (a global health non-profit organization), the Mozambique Ministry of Health (MISAU) and the World Health Organization (WHO), are planning to implement a strategy to reduce Missed Opportunities for Vaccination (MOV) in Mozambique during the last quarter of 2017. According to the Planning guide to reduce missed opportunities for vaccination, MOV include any contact with health services by a child (or adult) who is eligible for vaccination (unvaccinated, partially vaccinated or not up-to-date and free of contraindications to vaccination), which does not result in the individual receiving all the vaccine doses for which he or she is eligible. For our purposes, the focus will be only on children below two years of age.

The MOV strategy as described in the Methodology for the assessment of missed opportunities for vaccination will aim to demonstrate the magnitude and identify causes of these missed opportunities, followed by tailored health system interventions with the objective to reduce them, leading to an increase in vaccination coverage and timeliness of routine vaccinations.

While it is widely accepted that some of the missed children (commonly referred to as the 5 ^th^ child) have limited access to health services, previous studies reported in the Planning Guide suggest that a proportion of the missed children may already be accessing treatment and vaccination services. Unfortunately, for a variety of reasons, many health services continue to miss the opportunity to vaccinate these eligible children. Therefore, the MOV strategy emphasizes reducing MOV at the health facility level on a day-to-day basis and consequently increasing immunization coverage at existing vaccination sites (health centres, hospitals, outreach/mobile services). Furthermore, reducing MOV will also improve timeliness of vaccination, improve the efficiency of health service delivery in general, and promote synergies between treatment services and preventive programs at the health facility level. This is in line with other previous recommendations that have been made on the same to address this widely prevalent issue ^1^.

There has been an increasing momentum at global, regional and country levels to conduct MOV assessments in different settings. During the 2014 review of the Global Vaccine Action Plan, the Strategic Advisory Group of Experts on immunization (SAGE) recommended MOV studies based on evidence indicating a globally pooled prevalence of 32% ^2^. According to the Planning Guide, previous MOV assessments suggest several reasons for MOV linked to service delivery, vaccine shortages or other logistics barriers and demand-related barriers. That said, the findings tend to be country-specific and preclude standardized solutions ^2^.

### Study objectives

In spite of partner support to the EPI program in Mozambique, the program continues to under-perform by several measures. This study will assess the magnitude and causes of MOV in three provinces that have received varying degrees of vaccine delivery strengthening interventions (e.g. optimized vaccine supply chain) and short-term interventions (e.g. immunization campaigns). This study will allow for some comparisons by province or type of barrier (e.g. vaccine shortages or other logistics barriers; the failure or inability of health providers to screen patients for eligibility; perceived contraindications to vaccination on the part of providers and parents).

*Key questions*

The MOV strategy answers three important questions:

a) How many opportunities for vaccination are missed at existing vaccination sites?
b) Why are opportunities for vaccination missed at the different vaccination sites?
c) What can be adjusted or done differently (e.g. policies, behaviours, structural or organizational changes) in order to eliminate MOV?

### Results measurement

a) Estimate the extent of MOV and examine different correlations between MOV and other explanatory and demographic factors;
b) Estimate the proportion of children with missed opportunities, the proportion of visits with missed opportunities and the number of eligible doses missed, by antigen;
c) Classification of MOV by cause (e.g. logistics-, care-giver- or health provider-related).

## Study design

This study will employ a mixed-methods approach, incorporating both qualitative and quantitative tools. A mixed-methods approach according to Creswell (2003) is one where researchers tend to base knowledge claims on pragmatic grounds (e.g., consequence-oriented, problem-centred, and pluralistic) ^3^. The researcher bases the inquiry on the assumption that collecting diverse types of data best provides an understanding of a research problem ^3^.

## Study population

### Population

a) Caregivers (mother or any other person responsible for the child) of children between the ages of 0–23 months;
b) Health workers (both preventive and curative department staff).

### Inclusion/exclusion criteria

The MOV assessment targets caregivers of children between the ages of 0–23 months who attend a clinic in the selected districts on the day of the assessment. Regardless of the reason for visiting the health facility, their place of residence or relationship to the child, all caregivers are eligible to be interviewed. The caregiver may be the person who gave birth to or adopted the child, or is otherwise taking care of the child, such as an aunt, grandmother, or father. Caregivers should be at least 18 years of age. The assessment also targets all health workers (both preventive and curative department staff) that work in the selected health facilities on the day of the assessment.

### Sample size

According to the Methodology Guide, a sample size of 600 caregiver exit interviews and 300 health worker questionnaires will be targeted in a purposive sample of 30 health facilities.

## Sampling criteria

### Provincial, district and health facility sampling

We have selected three out of the eleven provinces based on the EPI team’s perceptions of performance, reported coverage rates, variability of vaccine delivery interventions received and region/location (North, South, Central). The three selected provinces are: Niassa (Northern region), Zambezia (Central region) and Maputo (South).

On average, each province has about 15 districts. We will aim for two districts per province (N=6), selecting a well performing and a poorly performing district. The criteria for selection of districts will take into account the following indicators: administrative coverage, stock out rates and cold chain equipment functioning. Data from two electronic systems SELV and SISMA will be reviewed for a period of three months (April to June 2017). For the three indicators, the vaccination coverage (children completely vaccinated), stock out and fridge functioning will be analyzed. Good performing districts will be defined as having more than 80% of vaccination coverage, less than 10% of stock out and more than 95% of fridges functioning. Poorly performing districts will be defined as having less than 50% of vaccination coverage, more than 10% of stock out and less than 95% of fridges functioning.

In each of the six districts, we will select five health facilities (N=30). The health facilities will comprise of a mix of hospitals, health centres and clinics. We will assign an identification number to each health facility and apply a randomization technique.

### Participant sampling

At each health facility visited, the field team will conduct 20 sequential exit interviews and 6–10 Focus Group Discussions (FGDs) with caregivers. Field teams will be made aware of the following core principles:

a) Aim to conduct a fixed number of interviews at each health facility selected (‘quota sampling’):
  – 10 exit interviews with caregivers of infants 0–11 months old;
  – 10 exit interviews with caregivers of children 12–23 months old;
b) Aim to speak with caregivers attending the facility for different purposes, i.e. if possible, an equal mix of caregivers seeking immunization vs. other services;
c) Whenever possible, data collection should be spread across several different health facilities (for example, no more than 20 exit interviews in any one facility)
d) If smaller health facilities are selected, it may not be possible to interview up to 20 caregivers. In such instances, additional health facilities will be visited to make up the sample quota for each field team. In these situations, the additional facilities will have similar characteristics to the one originally sampled, e.g. size/patient flow, district of location.

The assessment will also target all health workers (both preventive and curative department staff) that work in the selected health facilities on the day of the assessment for interviews and FGDs. 6–10 FGDs for health workers will be scheduled. Each FGD will have 6 to 10 participants for effective dialogue to take place. For health workers, it might be difficult to mobilize an FGD if they are too few or are not available to all meet at the same time. In such instances, a few in-depth interviews may be considered.

Finally, a purposive sample of senior staff and health administrators at multiple levels of the health system will be selected.

## Methods

Table 1 presents an overview of the proposed tools for this study. The detailed tools are available from the methodology guide on the WHO website.

**Table 1.** Tools for assessment of missed opportunities for vaccination (MOV).

Tools	Type of data to be collected
1. Health facility exit interviews (interviewer-administered)	- socio-demographic information; - vaccination history (routine immunization) *; - awareness of opportunities for routine immunizations; - home-based record (vaccination card/health passport) availability and retention; - reasons for non-vaccination; and - quality of the vaccination service received.
2. Health worker knowledge, attitudes and practices survey (interviewer-administered)	Knowledge about vaccination, including antigens, immunization schedules, and contraindications to vaccination.
3. Focus group discussions (with caregivers and health workers)	Exploring causes of MOV, and potential solutions and barriers to implementation of proposed interventions.
4. In-depth interviews (with senior staff and health administrators)	Interviews will be conducted with individuals who are insightful or influential about the health facility or the community, such as the health facility heads or in-charges, directors, matrons and administrators. This facilitates triangulation with other data elements and provides information regarding the informants’ perception of the community and health facility dynamics affecting immunization services, informant support or opposition to immunization. Key informants will also be asked to explain health worker and caregiver behaviors/responses and suggest or validate/refute previously proposed interventions to reduce missed opportunities.
Brainstorming sessions	Present research findings to immunization stakeholders and formulate an action plan with proposed interventions.

* Vaccination status will be documented from the information on the home-based records or health facility registers, where possible. The data collection team will not rely on the caregivers’ recall if the required record has been lost or is otherwise unavailable. This is particularly important because the date of vaccination is critical in assessing MOV.

### Study procedures

***Facilitator workshop.*** Facilitators will meet 1–2 days prior to the beginning of field team training to finalize the remaining logistic details for the assessment (such as finalizing the list of sampled facilities, transport arrangements, training logistics) and to finalize the data collection tools.

***Training of data collectors.*** All data collectors will be centrally trained over a period of 4 days to prepare them for data collection.

***Enrolment process.*** All caregivers exiting the health facility with a child that appears to be between 0–23 months are eligible and will be asked to participate. Each potential participant will be pre-screened on age of child only. The interviewer will explain the process and seek for written consent to participate in the interview. The questionnaire will be administered to consenting caregivers of eligible children, or any other consenting adult member (defined as being ≥18 years of age) accompanying the child, who is knowledgeable about the mother’s background and the vaccination status of the child. If an adult is accompanied by more than one child, the interview will focus on the youngest child. Caregivers who attend the facility for a variety of purposes (i.e. immunization as well as other services) will be targeted. In situations where the EPI clinic is located separately from the outpatient department, the interviewers will move around to interact with caregivers exiting at different points of the facility complex. For the health worker interviews, all health workers (both preventive and curative department staff) working in the selected health facilities on the day of the assessment are eligible to be interviewed.

***Data collection.*** The MOV assessment is designed to be completed in less than 10 days, including training, data collection and preliminary data analysis. Preparation of field teams through training and pilot testing of the data collection tool will commence in November 2017. Field teams will spend a maximum of two days in each health facility. Data will be collected using an electronic data platform where each fieldworker will be given a tablet computer. Standardized paper interview questionnaires will be available for back-up purposes.

As outlined in the Planning Guide, field teams (interviewers and supervisors) will be drawn from MOH staff and other in-country immunization partners since they are knowledgeable about the EPI program. In addition, we will involve a few independent researchers with experience in conducting similar work. This assessment will utilize a three-person team for province.

### Data management and analysis

The use of an electronic platform for data collection will facilitate preliminary analysis immediately after data collection for presentation at the brainstorming and debrief sessions. All data on the electronic data platform will be exported into Excel, and then to *Epi Info*™ 7 for data cleaning, data management and analysis. Initially, a simple analysis of quantitative and qualitative data will be carried out. Preliminary results will consist mainly of simple frequencies and bivariate analyses of quantitative data and a preliminary interpretation of key themes of the qualitative data. The results will be presented at the opening of the brainstorming session with stakeholders. These analyses coupled with other anecdotal information from the field will be used to inform the development of an action plan to reduce MOV.

In the weeks following fieldwork, a more detailed analysis will be carried out once all the data are compiled. The detailed analysis will estimate the extent of MOV as well as examine different correlations between MOV and other explanatory and demographic factors. Estimates of the proportion of children with missed opportunities will be calculated, as well as the proportion of visits with missed opportunities and the number of eligible doses that were missed, by antigen. Other analyses may include home-based record availability and vaccine coverage by antigen. These analyses will be stratified using other explanatory variables in the dataset, as appropriate. Where possible, the results will be compared to other recent surveys in the country, including Demographic Health Surveys and Multiple Indicator Cluster Surveys.

Thematic analysis of data from the *qualitative* aspects of the assessment will be conducted and included in a final report, presenting the scope of interviews, observations, representative verbatim quotes and key conclusions. This thematic analysis will be conducted using standard qualitative data analysis software (Atlas TI 8.0). Where possible, community and key informant recommendations for reducing missed opportunities will be included along with other study conclusions.

***Data security.*** There will be minimal collection of personally identifiable information (PII); however, where necessary any electronic datasets with PII will be password-protected. All electronic databases will have PII removed before any off-site analysis. The tablets will automatically record the time of the interview as well as the location where the forms were completed. Supervisors will review all data collection forms for completeness and accuracy prior to submission to the Assessment Coordinator (GJ). Interviewers will upload data every evening, so that further data quality checks can be conducted by BM, SS, IO. To enable the best quality data entry, the data entry forms will be designed using value constraints and data checks to the extent possible.

## Ethical considerations

The Nacional de Bioetica (National Bioethics Committee) in Mozambique granted ethical clearance for this study (Reference number 1008). Furthermore, each of the provinces provided permission for the study.

Respondents in this assessment (caregivers and health workers) will be requested to provide written consent prior to the interview. They will be informed that participation is voluntary and optional and that their responses will in no way affect their ability to access services or threaten their employment in the case of health workers.

## Limitations

The MOV assessment process is subject to some limitations and biases inherent in the simplified sampling methodology and the convenience sample of all children available on the day of the study, including selection bias. Also, the health facility staff could modify their practices on the day of the assessment. To minimize the impact of any changes in practices, the administration of health worker KAP questionnaires will take place in the afternoon, after the vaccination clinic sessions for the day have been completed and caregivers should only be interviewed upon exiting the facility, after they have received the service. Similarly, the FGDs will be conducted with different health workers (at a different health facility) than those that completed the health worker questionnaires. The assessment results will need to be interpreted with caution, as the sample will not necessarily be nationally representative.

## Dissemination

Summary reports will be developed for each district and province. Preliminary results will be presented and discussed at brainstorming sessions and debrief meetings with immunization stakeholders. Results will also be reported to the district, provincial and national-level immunization program staff, using regular feedback and training meetings. Results may be presented at professional meetings, and national, regional or international scientific meetings. To accelerate cross-country and peer-to-peer learning, results from the MOV assessment be written up for publication in peer-reviewed journals.

## Conclusion

A concerted effort to reduce or eliminate missed opportunities, especially by addressing persistent barriers, could result in increased vaccine coverage by up to 30%. In addition to using data to improve local vaccination coverage in Mozambique, the findings could contribute to a better understanding of MOV in similar settings.
