# Supplementary material for: Reducing missed opportunities for vaccination in selected provinces of Mozambique: A study protocol
Source: Gates Open Res. 2017 Nov 6;1:5. [Version 1] doi: 10.12688/gatesopenres.12761.1 (PMC5841573; doi:10.12688/gatesopenres.12761.1)
Supplement: Peer review history

# Peer review: Referee response for version 1

**DOI:** 10.21956/gatesopenres.13821.r26120

**Published:** 2017-11-29

**Authors:** Felicity T. Cutts, Dale A. Rhoda

**Affiliations:** 1 London School of Hygiene & Tropical Medicine, London, UK; 2 Biostat Global Consulting, Worthington, OH, USA

**Detailed comments**

 This research protocol is based on resources from the recently developed WHO strategy for reducing missed opportunities for vaccination (MOVs) ( http://www.who.int/immunization/programmes_systems/policies_strategies/MOV/en/), and in particular the MOV Methodology. The protocol is clearly written and follows the WHO guidance closely. It is based on formative research which gives priority to public health action and not solely to more technical aspects of the assessment of the prevalence of MOVs. As such it should be expected to lead to a participatory process of investigation of the importance of MOVs and the development of strategies to reduce their occurrence. The WHO strategy is tailored to in-country public health professionals with limited time and/or research experience, aiming to provide field-friendly, practicable tools. This study will provide further experience with the use of the tools and contribute to knowledge of how assessment of MOVs and their causes can lead to effective public health action. It should be noted, however, that the study is not a rigorous statistical measurement of the prevalence of MOVs.

**Rationale and objectives**

 The introduction summarizes key reasons why assessment of MOVs is considered important at the global level and notes that vaccination coverage appears to have stagnated in Mozambique. Previous work on MOVs in Mozambique is not, however cited, and the authors are encouraged to review the relevant literature from their country.

 In the abstract and conclusions, the authors suggest that by avoiding MOVs, coverage could be increased by up to 30% but provide no data on which to base this claim. Indeed, given that according to WHO-UNICEF estimates, coverage of 3 ^rd^ dose of pentavalent vaccine is 80%, it is difficult to see how coverage could increase by another 30%!

 The study objectives, key questions and results measurement indicate that the authors wish to measure the frequency of MOVs in 3 provinces; to assess variation in MOV frequency according to variation in potential contributing factors such as stockouts; and to classify the causes of MOVs. Most importantly, the study aims to determine what can be done differently to reduce MOV occurrence.  

 The study design does not, however, lend itself to meeting all these objectives in a statistically rigorous way. This should be made clearer in the protocol – the WHO methodology sacrifices statistical rigour for programmatic utility and while the trade-off is likely to be appropriate, it needs to be clearly acknowledged.

**Study design**

 These health-facility-based studies, conducted in a purposive sample of facilities in only a few districts on a single day per facility, give an indication of how common MOVs are on the day of the visit as well as qualitative insights into the causes of MOVs, but are not designed to give a representative estimate of MOV frequency.  

 By contrast, data from high-quality household surveys can be analysed to assess the frequency of one type of MOV which is non-simultaneous vaccination – the failure to administer all vaccines for which the child is eligible at a visit when at least one vaccine is administered (Jani et al., 2008, Cutts et al., 1989). The 2015 update of the WHO coverage survey guidelines provides information on how to analyse coverage surveys (including multiple-indicator surveys such as DHS or MICS) to obtain estimates of the frequency of MOVs (and gives detailed guidance on different potential definitions of MOVs). Unfortunately, it appears that there have been no recent national vaccination coverage surveys in Mozambique – the most recent one used in the WHO-UNICEF estimates was the 2011 DHS (see section 4.6 “coverage survey data” on http://www.who.int/immunization/monitoring_surveillance/data/en/) – thus this approach may not be feasible at national level at present. The authors could, however, look for data from subnational surveys that could be re-analysed to measure the frequency of MOVs at the population level.

**Details of study methods**

 The study methods follow closely the WHO guidance.

 It is unclear how the authors decided on just 2 districts per province. The choice of districts is said to be based on those with less than 50% admin coverage, and those with more than 80% coverage as well as indicators of cold chain and vaccine management. They do not provide information on the electronic data systems which provide these data for selection of districts. How do they define fully vaccinated – usually this is not an indicator which is included in routine reports. Are there districts having less than 50% pentavalent 3 coverage? According to the WHO website ( http://apps.who.int/gho/data/view.other.immu-admin2-dtp3?lang=en) the lowest district coverage is just over 60%.

 Sample size – as recommended by WHO, they aim for 600 exit interviews and 300 health worker interviews at 30 health facilities. However, this does not take into account their desired aim of comparing MOV frequency with various barriers in the different provinces. Indeed, the issue of how to calculate sample size (and how to calculate confidence intervals when analysing data) is difficult in the absence of a true probability sample and it might be best to accept that this is a semi-quantitative study that does not aim to provide statistically valid results. 

 The authors note the potential problems that may occur if there are very few children attending the facility on the interview day. An alternative sampling scheme whereby facilities are selected using probability proportional to estimated size (where size is average patient load per day or per month, depending on data availability) rather than simple random sampling could help to avoid this problem.

 The authors do not specify whether facilities will only be visited on days when vaccination is scheduled or irrespective of vaccination scheduling. In many countries, health facilities do not offer vaccination daily but only schedule one day per week or per month etc. for vaccination. The value of including hospitals is unclear – do hospitals in Mozambique provide vaccination services? If so, more information about how work would be organized in hospitals would be helpful. What is the difference between a health facility and a clinic? Will outreach sites be included? Outreach is often done to more remote areas, and these are areas where MOVs may be particularly important as it may be several weeks before the next health service contact, thus leaving children at risk of disease for longer.

 The need for 300 health worker interviews is not explained. This component of the study is likely to be time-consuming for data collection and data analysis and yet many of the health workers interviewed are unlikely to work directly with vaccination services. Perhaps one or two questions about referral guidelines for vaccination of children attending primarily for curative care could be asked as part of key informant interviews at facilities instead. 

 This protocol, and the WHO guidelines, do not include the collection of retrospective data on MOVs, nor any assessment of the quality of data recording on home-based records (HBRs) and facility registers. It would be straightforward to add these components to the study and need not prolong the duration of data collection (especially if fewer health worker interviews are conducted). For children with HBRs, dates of all vaccinations ever received can be recorded, allowing analysis of past MOVs which would not be subject to any potential bias from the presence of the study team on the day of interviews (and WHO has software pre-coded which can facilitate this). For these same children, dates of vaccination can also be extracted in the afternoons from facility registers, and compared with the dates on the HBRs to assess the quality of data recording – a type of data quality self-assessment. If more time is available, facility registers could be a rich source of data for a larger sample, potentially selected randomly, which would allow assessment of the frequency of MOVs at that facility over a period of time not just the day of the interview and would be more likely to provide statistically meaningful results. It would be very helpful to pilot such additions and determine the time and resources needed to add them to the WHO Methodology. 

 The section on selection and training of data collectors is short. It can be very helpful to include health workers from other districts or provinces in the data collection as they will gain insights into MOVs by participating in the assessment. Training should include enough time in the field to determine how to organise work at the facility, in order to be able to conduct sequential interviews without making mothers wait too long and without missing those who may leave via multiple exit points. Work at various types of facilities should be included in the practical training. There is no detail on training for focus groups which require different skills from those for exit interviews.  

 The protocol and WHO guidance says that the study should be complete in 10 days including training and analysis. This appears to be unduly short and likely to jeopardise data quality. It would seem wise to extend this, at least to allow additional time for training, 10 days for data collection, and additional time for data cleaning and analysis including, ideally, analysis of previous MOVs as well as those on the day of the visit.There will be a lot of qualitative data collected from all these interviews and focus groups, that take substantial time to compile and interpret.

**Datasets**

 Which electronic platform will be used for data entry?

 If both paper and electronic data collection are used, how will discrepancies be resolved? Or will paper forms only be consulted if data are lost from the electronic platform?

 For analysis, the authors might like to review the guidance in the WHO coverage survey manual on calculation of the frequency of MOVs using different definitions and, if interested, contact WHO for further information and guidance on appropriate software. 

 The authors aim to measure the proportion of MOVs occurring on the day of the visits but do not define clearly how this will be done – these analyses are not as straightforward as might initially be thought. First, definitions of eligibility for each vaccine-dose combination is needed, e.g. what is the minimum age that will be accepted for 1 ^st^ dose pentavalent vaccine – exactly 42 days or a few days before and if so, what lower age bound is acceptable? What will be done if the date of birth has been entered incorrectly on the HBR or register? For intervals between doses, likewise, will there be any flexibility below the recommended 4 weeks (28 days)? What, if any, contra-indications will be considered valid contra-indications to each vaccine? What is the national policy for vaccinating children who do not present with a home-based record and cannot be traced in the register. These details need to be worked out and documented before the analysis.

 The authors propose further analyses that are beyond the remit of this formative research. The study is not designed to measure coverage hence “analysis of vaccine coverage by antigen” is not meaningful –a convenience sample of 10 children in each age group at purposively selected facilities on one day does not represent coverage. It is not meaningful to try to compare results from this sample with those from surveys such as DHS or MICS. It is definitely helpful to analyse DHS and MICS data to obtain population-based measures of MOVs but these are not comparable to this sample. 

 Overall, this should be a useful study that helps to generate important discussion of strategies to reduce MOVs and address other barriers to vaccination which the interview results will suggest. It could be substantially enriched at relatively little marginal cost by including the assessment of past MOVs and the quality of data recording at the health facilities visited. It is important to remember that this is formative research and not try to use the data as if it were from a statistically rigorous sample.

We have read this submission. We believe that we have an appropriate level of expertise to confirm that it is of an acceptable scientific standard.

# Peer review: Referee response for version 1

**DOI:** 10.21956/gatesopenres.13821.r26052

**Published:** 2017-11-20

**Authors:** Robert Steinglass, Rebecca Fields

**Affiliations:** 1 JSI Research & Training Institute, Inc., Arlington, VA, USA

- Please provide more information on the selection of data collectors, particularly the qualifications needed
- More information is needed on the method for the brainstorming sessions that take place after the assessment but are considered integral to the MOV assessment methodology. At what level do they take place (national or also subnational); who should be involved—is it just “immunization stakeholders” when there are other stakeholders (particularly at subnational level) who have important roles to play in reducing MOV; what is the process.  Such information is vital to ensuring that the real purpose of the assessment – namely to reduce MOV – is served.
- Abstract: Is the expected 30% coverage increase in relative percent or absolute percentage points.
- Introduction: Make it clearer that you are referring to valid (not invalid) contraindications.
  32% prevalence of MOV needs a little more clarification for it to be understandable.
  Mention the limitation that health facilities that do not offer vaccination services (even though they could) are not included in the methodology and also that mothers who do not visit health facilities are excluded. 
- Sampling criteria:  “less than 10% of stockout” is not understandable.
- 6-10 FGD at EACH health facility is probably highly unlikely to implement, given the size of the health facility staff, assuming that a focus group is understood to be different than an interview with a single individual.
- It would also be useful to interview staff responsible for managing the vaccine supply chain to gain a possible additional perspective.
- Study procedures: Be prepared with rules when the adult is not in fact knowledgeable about the vaccination status of the child. Be careful to recruit interviewers and supervisors from staff who are not directly responsible for the program in the sampled health facilities. They should also be able to speak the local language.
- Data management and analysis: Given their lower power and different geographic focus, it is unclear how these results can be compared to large recent surveys in the country, including Demographic Health Surveys and Multiple Indicator Cluster Surveys.
- Limitations: This statement, affecting the methodology, was not at all clear earlier: “The FGDs will be conducted with different health workers (at a different health facility) than those that completed the health worker questionnaires.”
- Conclusion: Is the expected 30% coverage increase in relative percent or absolute percentage points?

We have read this submission. We believe that we have an appropriate level of expertise to confirm that it is of an acceptable scientific standard.
